# Brain Organoids: Emerging Platforms for Modern Neuroscience

**DOI:** 10.3390/brainsci16040427

**Published:** 2026-04-19

**Authors:** Lian Wang, Liwei Mao, Qing Cao, Xuemei Zong

**Affiliations:** 1 Department of Neurology, Institute for Cerebrovascular and Neuroregeneration Research (ICNR), Louisiana State University Health Sciences Center, 1501 Kings Highway, Shreveport, LA 71103, USA; wanglian9369@hotmail.com (L.W.); 15951911332@163.com (L.M.); 2 Department of Neurology, Medical College of Georgia, Augusta University, 1120 15th Street, Augusta, GA 30912, USA; 3 Department of Neurology, David Geffen School of Medicine, University of California, 710 Westwood Plaza, Los Angeles, CA 90095, USA

**Keywords:** brain organoids, 3D culture systems, neural development, disease modeling, bioengineering, organoid transplantation

## Abstract

**Highlights:**

**What are the main findings?**

- Brain organoids recapitulate key aspects of human brain development and disease, enabling human-relevant in vitro modeling.
- Advances in bioengineering and culture technologies improve structural organization, cellular diversity, and functional readouts of organoid systems.

**What are the implications of the main findings?**

- Organoid platforms support mechanistic studies and facilitate drug discovery and disease modeling in neuroscience.
- Improving maturation, reproducibility, and ethical frameworks will be essential for advancing organoids toward translational applications.

**Abstract:**

Brain organoids represent three-dimensional structures that allow for human-specific studies in brain development, pathology and therapeutics. These self-organizing systems, formed through the differentiation of human pluripotent stem cells, can mimic important cellular and molecular events of brain development and therefore serve as a platform for the investigation of neurodevelopmental and neurodegenerative diseases, brain injuries, and tumorigenesis. Although brain organoids show promising perspectives in the study of human physiology, existing brain organoid platforms are hindered by issues of under vascularization, immaturity and protocol variability. Nevertheless, the rapid development of new bioengineering, microfluidic and multi-omics tools and approaches allows us to overcome existing problems and increase the physiological significance of these organoids. Brain organoid transplantation and functional studies further enhance the applications of brain organoids in drug screening, disease modeling and personalized medicine. Here, we provide an overview of recent developments in the field of brain organoid cultures, functional characteristics and translational applications.

## 1. Introduction

Exploring human brain development and neurological disorders has been hampered by the shortcomings of established animal models and two-dimensional (2D) cell culture systems. Recent innovations in stem cell biology and three-dimensional (3D) culture methods have allowed researchers to develop brain organoids, self-organizing multicellular structures formed by human pluripotent stem cells (hPSCs) that mimic many of the cellular and molecular processes involved in human brain development. Brain organoids represent an innovative approach in terms of exploring human brain development and modeling disease pathogenesis and therapeutics in vitro.

Brain organoids comprise different region-specific models, ranging from cerebral organoids (COs) to resemble the forebrain, cortical organoids for simulating neocortical structures, and other structures mimicking midbrain, hindbrain, cerebellum, and hypothalamus development. Organoids allow us to explore some important processes related to early neurogenesis, including proliferation and migration of neural progenitors, formation of neural networks, and synaptogenesis. Although they cannot simulate complex stages of brain development or the establishment of extensive brain connectivity, they can be regarded as valuable tools for studying brain development both in cellular and certain circuit-related aspects. Applications of brain organoids are diverse and include investigations into neurodevelopmental disorders, neurodegenerative diseases, brain tumors, injuries, and other pathological conditions. In general, they capture some characteristics of these diseases but do not reproduce full pathology and can be used to provide insights about the underlying mechanisms. Moreover, brain organoids can serve as tools for drug screening, gene therapy development, and precision medicine. While many advances have already been made, the current limitations should be acknowledged. Variability across experimental procedures, limited vascularization, poor maturation of neurons, and lack of extensive connectivity can be regarded as major concerns. Nevertheless, several novel approaches, including assembloids, bioengineering, and genome editing, show promise of overcoming the current limitations.

**Figure 1:**
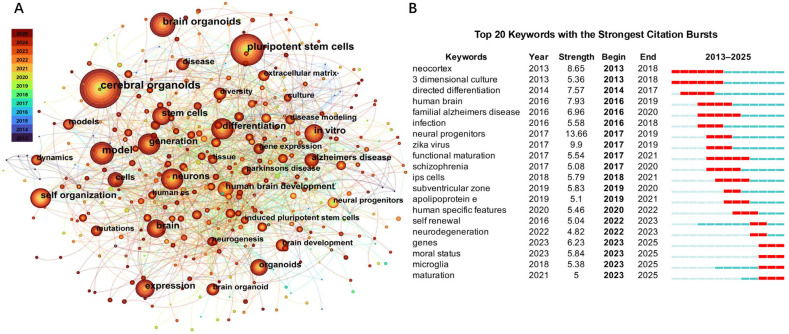
Network visualization of the co-occurrence of keywords and burst citations in the literature on brain and cerebral organoids. (**A**) Network of keyword co-occurrence in papers on brain and cerebral organoids from 2013–2025. Every node represents one keyword, where the size of the node is positively correlated with the number of occurrences of that keyword and the thickness of the edge is positively correlated with the intensity of the co-occurrence. Colors represent the average publication year of each keyword, with cooler colors (blue/green) indicating earlier years and warmer colors (yellow/red) indicating more recent years, illustrating the temporal evolution of research themes across the field. (**B**) The top 20 keywords with citation burst activities from 2013–2025. “Strength” refers to the intensity of the citation burst, “Begin” and “End” refer to the beginning and end year of the citation burst period, respectively. Red bars on the timeline represent years with citation bursts and light blue bars represent years without citation bursts.

In the present paper, we will provide a brief overview of brain organoids, paying special attention to advances in culture methods, properties of organoids, and clinical applications. In addition, we will discuss potential solutions for addressing the existing limitations of brain organoids. We will also analyze recent trends in brain organoid studies through bibliometric analysis of articles published between 2013 and 2025 with the keywords ‘brain’, ‘cerebral’ and ‘organoids’, using CiteSpace (version 6.4.R1, Drexel University, Philadelphia, PA, USA) (Figure 1).

## 2. Brain Organoid Culture and Engineering

**Figure 2:**
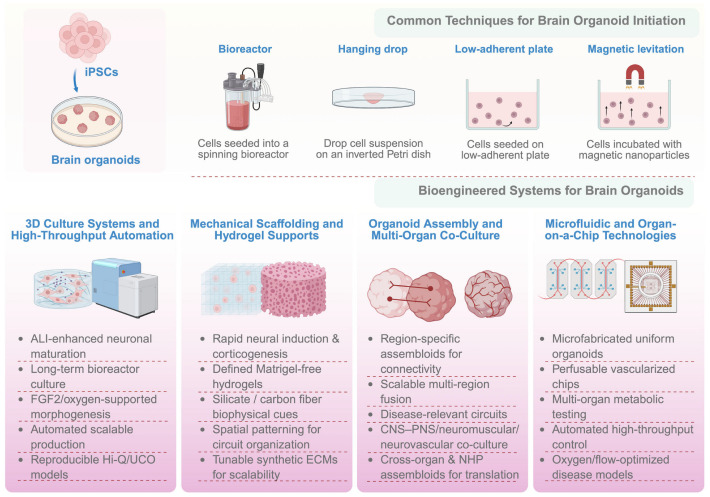
Strategies for engineering brain organoids. Engineering of brain organoids employs a combination of classical and advanced bioengineering methods to recreate elements of brain structure and function in a more accurate way. Popular methods of brain organoid formation include suspension culture using bioreactors, hanging drop aggregation, low-adherent plate seeding, and magnetic levitation, which are all aimed at inducing self-organization of stem cells into a three-dimensional structure. New engineering developments in brain organoids use biointegrative devices to increase accuracy and scalability; however, the efficiency may differ based on the design and growth conditions. These devices include 3D culturing systems, automated bioreactors, mechanical scaffoldings and hydrogels for tissue engineering, multi-region cocultures to model inter-regional connections, and microfluidics or organ-on-chip devices. The arrow indicates the transition from iPSCs to brain organoids.

To present an integrative picture of the major techniques used in organoid studies, this review focuses on the major experimental platforms used from the beginning to advanced stages of brain organoid experiments (Figure 2). The techniques discussed include not only classical culturing technologies but also advanced engineering techniques that make it possible to control organoid development and analyze their functions. In this context, organoids can be generated using basic techniques such as choosing the type of stem cells and methods for inducing them to develop along neural lineages and basic 3D culturing conditions, with other engineering techniques being added to this process.

### 2.1. Cell Sources and Neural Induction

Brain organoid development has concentrated on the efficiency, reproducibility, and maturity of in vitro neurogenesis. The most applied neural induction procedure utilizes dual-SMAD inhibition by employing Noggin and SB431542 and modulating the Wnt pathway with CHIR99021. In this way, neurogenesis is achieved from adherent hPSCs without using embryoid bodies and neuroepithelial organoids containing many neural cell types can be obtained which simulate brain development in early stages [1]. The use of region-specific patterning molecules can increase organization of the tissue and produce cortical-like structures, although functional maturation and circuit integration remain rather unpredictable among different protocols [2]. Electrophysiological activity was demonstrated in brain organoids, but these results are highly dependent on experimental procedures and cannot be easily reproduced in other labs [3]. To address the problem of low scale up, researchers have explored alternative ways to derive organoids through stem cells of different origins and engineering-based protocols. Induced pluripotent stem cells (iPSCs) can be collected non-invasively and used to generate cortex organoids. Techniques like 3D bioprinting and semi-guided protocols help achieve precise spatial arrangement and diversity but come with increased unpredictability [4]. The pre-inducing conditions, such as activation of fibroblast growth factor pathways, also affect the ability to form neuroepithelial tissue [5]. More simplified culture models have been introduced to streamline the process of producing organoids, though at the cost of their maturation and cell composition [6]. Despite being able to produce specific structures spontaneously, brain organoids demonstrate less precision in terms of spatial arrangement compared with physiological brain structures [7]. There are also cross-species systems that are helpful in obtaining information about brain development mechanisms but which should be interpreted cautiously when trying to apply the gained knowledge to humans [8]. However, there are other ways brain organoids can be used in addition to developmental studies, including the study of tumors, neurovascular interactions, and personalized medicine trials [9,10]. Thus, the recent advancements in cell source and neural induction methods have allowed for the reaching of significant success in creating highly controllable and versatile brain organoids for various purposes. Protocols that maximize reproducibility and scalability tend to sacrifice biological accuracy and vice versa.

### 2.2. 3D Culture Systems and Automation

One of the advances that increases controllability and even somewhat improves the physiological relevancy of the organoid brain model is the use of three-dimensional cultures. Air–liquid interface (ALI) platforms specifically allow for neuronal maturation by improving the survival rate and axon growth, which in turn allows the creation of nerve tract-like structures, allowing for functional interactions in co-cultures [11]. Organoids produced from cortical organoid slices using the ALI technique are capable of developing even more complicated cellular architectures and even spontaneously occurring network activities; however, the relation of in vitro circuits in these models to those seen in vivo is not always accurate [12]. Some bioreactors have been created to help in the development of stable and durable tissues. One example would be the SpinΩ system, which can allow for the scaling up of cultures as well as the region-specific culturing of organoids, while suspension and rotational culture techniques contribute to improved tissue formation [13,14]. Gravity-defying bioreactors could reportedly contribute to increased yield and diversity; however, these are hard to reproduce and access [15]. Other techniques include automation and microfabrication, which can improve scalability and standardization of procedures. Automated multiwell plates as well as custom-made 3D-printed platforms increase control over the size and reduce manual interference, though these require additional equipment [16,17]. The main problems facing current technologies are the variability of different platforms and the incomplete differentiation of neuronal cells, both hindering the accurate simulation of complex processes happening in vivo. All of the above technologies combined help create scalable and versatile experimental settings. From the available platforms, bioreactor and automated multiwell solutions are thought to provide better results in terms of scalability and reliability.

### 2.3. Scaffolds and Hydrogel Systems

Hydrogel-based scaffolds are widely used to improve the structural organization and developmental control of COs. Hydrogels containing chemically defined factors facilitate early neural patterning and the development of neuroepithelial constructs that allow region-specific differentiation and neuronal activities [18]. In contrast to undefined scaffolds like Matrigel, chemically defined hydrogels can be easily reproduced and are more precise in composition; however, their capacity for complete recapitulation of natural extracellular environments is limited [19]. Apart from biochemical guidance, mechanically defined materials also serve as a source of physical cues affecting tissue organization and neuronal maturation. Fiber-based materials facilitate better cortical organization and lineage-specific differentiation, including midbrain dopaminergic neurons [20]. The next level of design, which involves spatial patterning or gradients, allows for better organization and more effective regional specification of neuronal tissue; however, the complexity of fabrication processes and scalability limitations are significant concerns in such approaches [21]. Moreover, extracellular matrix (ECM)-mimetic materials can stimulate certain cellular functions, such as synaptogenesis and neuronal connectivity [22,23]. Mechanical properties of certain materials, for example, in compressed or structured systems, stress the importance of physical factors in neuronal maturation [24]. The recent achievements in biofabrication have allowed scientists to create more complex and scalable scaffold systems, including synthetic ECM analogs and microfiber materials that increase the viability of tissue due to improved nutrient and oxygen flow [25]. Despite some challenges in balancing biological complexity and reproducibility, various scaffold materials enable the creation of more complex and functional neural organoids. It should be noted that, despite having better reproducibility and tuning capacity than Matrigel, chemically defined hydrogels still lack the biochemical complexity of the extracellular environment to support tissue maturation. Conversely, ECM-rich or hybrid systems better support functional development but introduce variability and batch dependence.

### 2.4. Organoid Assembly and Co-Culture

The assembly of region-specific organoids into structured assembloids provides a platform to investigate inter-regional interactions and aspects of circuit formation. Co-culture systems combining distinct brain regions support axonal extension, neuronal migration, and coordinated activity patterns, enabling the study of structural and functional connectivity in vitro [26]. However, these systems primarily approximate early developmental processes and do not fully recapitulate the complexity or precision of in vivo neural circuits. Engineered assembloids have also been applied to disease modeling by capturing selected features of circuit dysfunction. Patterned cortical assembloids reproduce aspects of regional gene expression and developmental abnormalities, while cortico-striatal and midbrain-striatal assembloids support the study of synaptic integration and dopaminergic signaling relevant to neurodevelopmental and neurodegenerative disorders [27]. These models help identify disease mechanisms, although their predictive value varies and is often rather low. In addition to brain-related systems, the use of multi-organ assembloids involves other tissue types, such as muscles or blood vessels, to model system interaction. Such models help study neuromuscular and neurovascular connections, as well as the effect of systemic metabolism on neural function [28,29]. Multi-system integration also includes non-neural tissues, for example, liver spheroids, thus helping explore the role of systemic factors in neurotoxicity [30]. Increasing system complexity raises some issues related to maintaining the consistency and reliability of the connection between organs. Non-human primate and human-based assembloids offer a chance to study species-specific brain developmental processes. Such models allow for the exploration of neuronal migration and the ratio of excitation and inhibition in the brain, which can contribute to further comparative analysis [31,32]. Scaling, standardization, and ethical aspects become relevant when working with animals. The implementation of assemblies and co-cultures expands the possibilities provided by brain organoid models. Thus, researchers can study inter-region connections and multi-system interactions using this approach in vitro. However, an increase in the level of system complexity implies some limitations related to reproducibility, scalability, and interpretation. More reliable results can be achieved using simpler co-cultures, while complex assembloids can better represent interactions within multi-systems.

### 2.5. Microfluidic and Organ-on-Chip Systems

Microfluidic technology and organ-on-chip models have recently emerged as promising tools to improve reproducibility and environmental control of CO cultures. Static microfluidic models based on microwell/micropillar platforms promote reproducible formation of embryoid bodies, minimizing variability related to handling [33,34]. While these models can greatly improve the early stages of homogeneity in COs, they fail to provide effective solutions to improve the complexity of late-stage organoids. Dynamic microfluidic platforms take the benefits of static systems one step further and introduce new features of perfusion and biomimetic environments. Microfluidic devices that use perfusion improve nutrient supply, eliminate necrotic core formation, and partially mimic vascular organization of developing organs, resulting in improved survival and patterning [35]. Multiple organ chips also allow for the investigation of communication between different organs and for the study of systemic responses to drugs [36]. Although microfluidic devices significantly improve the environment of organoid cultures, the presence of fully developed vascular structures and the mimicking of the physiological processes of blood flow and BBB formation remains challenging. To make microfluidic models more scalable and controllable, automated fabrication systems and closed loop designs were developed for microfluidics [37]. While these systems improve the process of CO culture, they remain complicated and costly. Additionally, the process of culturing requires a significant amount of maintenance, which complicates experiments. The advantages of microfluidic systems have been successfully used for studying different biological functions and pathologies in vitro. Studies on disease modeling using microfluidic systems include investigation of neurotoxic effects, the study of behavior of tumor cells, and neurotransmission [38,39]. Due to improved flow control, imaging in real time is more efficient, and manipulating the microenvironment in the culture chamber becomes more accessible in comparison with conventional techniques. Unfortunately, the function of brain-derived COs often requires electrophysiological analysis; however, its outcomes depend greatly on system design and experiment settings. The described technologies provide improved control over organoid environment and opportunities to scale up organoids’ dynamic modeling of brain development and disorders. Different configurations have varied robustness; however, the simpler configuration tends to be more reproducible, while a more complex setup allows the reproduction of certain aspects of in vivo processes.

## 3. Functional Properties of Brain Organoids

**Figure 3:**
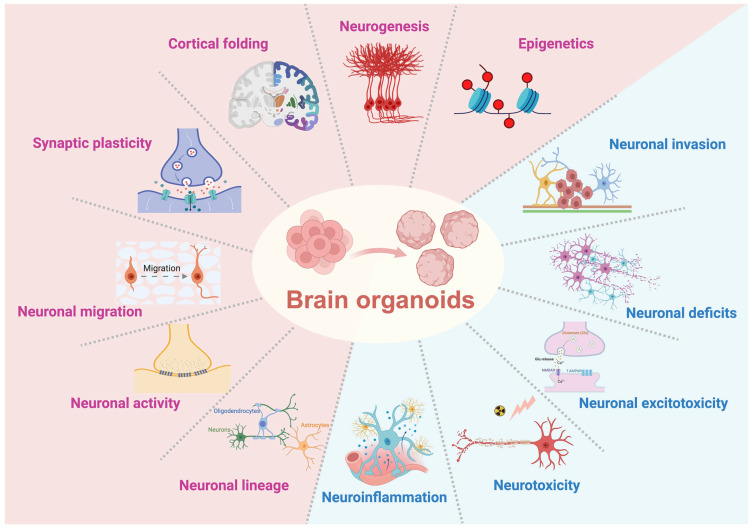
Functional properties modeled by brain organoids. Brain organoids recapitulate key aspects of human brain development, including cortical folding, neurogenesis, neuronal lineage specification, migration, synaptic plasticity, and neuronal activity. In addition, they capture pathological features such as neuroinflammation, neurotoxicity, excitotoxicity, neuronal invasion, and functional deficits, as well as regulatory influences including epigenetic modulation. These features highlight the ability of brain organoids to model both developmental processes and disease-related phenotypes, supporting their application in mechanistic and translational studies. The arrow indicates the transition from iPSCs to brain organoids.

Brain organoids exhibit a range of functional features that reflect key aspects of human brain development and disease. In addition to generating diverse neuronal and glial cell types, these systems display measurable properties, including neuronal activity, network formation, and cell-type-specific responses to genetic and environmental perturbations. They also enable modeling of disease-associated phenotypes, such as altered neuronal function, disrupted connectivity, and pathological cellular responses. However, these features primarily represent early developmental states, and their ability to capture mature, system-level brain function remains limited. The following sections summarize these functional properties and highlight their relevance for the study of neurodevelopment and neurological disorders (Figure 3).

### 3.1. Neurogenesis

Neurogenesis in COs recapitulates key features of early human brain development, providing a platform to study progenitor dynamics, lineage specification, and molecular regulation. Radial glial cells, including basal radial glial, play central roles in neurogenic output through regulated divisions and Notch-dependent fate decisions, supporting self-renewal and neuronal differentiation in a manner broadly consistent with in vivo development [40,41]. Evolutionary processes are recapitulated in the context of organoid-based modeling, where human-specific factors can promote progenitor proliferation and metabolism, thus enabling cortical expansion [42]. Intracellular mechanisms governing neurogenesis in organoids include transcriptional and post-transcriptional control, mitochondrial dynamics, and epitranscriptomics regulating cell cycle dynamics, protein production, and progenitor survival [43,44,45]. Regulation by methylation of RNAs further includes lineage diversification in various brain structures, such as hypothalamic neurogenesis [46]. On top of intracellular mechanisms, extracellular control of neurogenesis also exists via signaling pathways involving growth factors and GPCR receptors, as well as other developmental signals like Slit/Robo and Notch signaling that regulate progenitor proliferation, corticogenesis, and the ratio between direct and indirect neurogenesis [47,48,49,50]. Another novel technique, based on controlled neurotrophic factor release, provides another level of bioengineering modulation of neurogenesis [51]. In addition, brain organoids have already been applied for modeling neurodevelopmental and neurodegenerative disorders associated with aberrant neurogenesis. Indeed, gene manipulations impacting protein homeostasis, mitochondrial dynamics, or any signaling pathways can disrupt proper neurogenesis and lineage diversification, as exemplified in neurodevelopmental or neurodegenerative disease models [52,53]. This opens an opportunity for the performance of pharmacological screens to identify therapeutic interventions for restoring neurogenesis and cell fate specification [54]. Organoid neurogenesis thus represents a practical approach to studying the relationship between molecular regulation and developmental consequences.

### 3.2. Cortical Folding

The generation of cortical folding in brain organoids is driven by the coordinated interaction of the multiple regulatory mechanisms involved in progenitor proliferation, spatial orientation, and tissue expansion. Human-specific genetic regulatory programs mediate such processes; for example, ARHGAP11B and NOTCH-related regulators promote basal and apical progenitor expansion, thus contributing to cortical expansion and folding [55,56]. Other regulators, such as growth factors and proneural transcription factors, impact progenitor proliferation and the balance between proliferative and neurogenic divisions, thereby influencing the pattern of cortical growth [57,58]. Alterations in the corresponding pathways, such as PTEN deficiency, result in abnormal progenitor expansion and folding-like patterns [59]. Not only are intrinsic genetic programs involved in cortical folding, but extracellular matrix composition and cell adhesion molecules also play important roles in determining progenitor spatial location, movement, and tissue stability [60,61]. Biomechanical effects associated with differential tissue growth and cortical tension have also been proposed as drivers of cortical folding, where variations in thickness and expansion cause surface buckling due to physical stress [62,63]. Overall, the processes of cortical folding in humans appear to involve coordinated action of chemical signals and mechanical forces. The bioengineering approach can be used to create models of human cortical folding in vitro. For instance, a folding-like patterning can be achieved through the use of gradients of morphogens or external mechanical stress, providing experimental means for studying gyrification and its pathological forms [64,65]. The folding of the cortex in organoids is a result of the complex interaction between genetic regulation, mechanical forces, and spatial patterning in human corticogenesis.

### 3.3. Synaptic Plasticity

Synaptic plasticity is one of the main functional characteristics of neural circuits involved in learning and memorization. Brain organoids offer a valuable in vitro system for studying the process of development and the adaptation of neuron connections. The introduction of inter-organoid connectivity or region-specific assembloids improves the network activity and short-term plasticity due to more intensive connectivity of the circuits [66,67]. However, these models offer an opportunity for investigating the human specificities of synaptic regulation within a relatively controlled context, though their activity levels cannot be compared with the mature ones found in vivo. Brain organoids are used for developing the models of disease-related disturbances in synaptic plasticity. Genetically induced defects in synaptogenesis, protein synthesis, or cell architecture result in impaired connectivity and decreased functionality in brain organoid models of neurological and epileptic conditions [68]. These models help establish the connection between the molecular changes and resulting physiological consequences, allowing mechanistic investigation of synaptopathies. The activity-dependent synaptic remodeling in organoids involves protein synthesis in the neurons and signaling pathways. Intracellular pathways related to local protein synthesis influence the synaptic responsivity, while pathway modulation, e.g., in the Hippo signaling pathway, improves the synaptic functionality through AMPAR-dependent mechanisms [69,70]. Thus, brain organoids offer a model of synaptic plasticity based on the activity-dependent remodeling of the neural circuits.

### 3.4. Neurotoxicity

Brain organoids offer a human-relevant model system to explore the cellular and molecular processes involved in neurotoxic responses following environmental and pharmaceutic exposure. Indeed, several toxic agents, including heavy metals, air pollution, and nanoparticles, are capable of negatively influencing brain organoids’ capacity to develop neurons through mechanisms including neuronal cell death, disruption of neurogenesis, and alteration of cerebral cortex organization [71,72]. Such responses are often associated with altered mitochondrial function, oxidative stress, and perturbation of critical signaling processes such as Wnt signaling, suggesting the existence of common mechanisms among neurotoxic processes. Moreover, brain organoids have become extensively used in drug-induced neurotoxicity investigations. In particular, exposure to various drugs, ranging from anti-depressants to neurotoxins and chemotherapy drugs, causes a reduction in synaptic protein levels, neurite formation deficits, and neuronal population-specific toxicity [73,74,75]. These results confirm the value of organoids in relating molecular/cellular alterations to dysfunction, although dose dependence and variation between models is also important. In addition to environmental and pharmacological factors, organoids enable the study of genetically driven neurotoxicity. Disease-associated mutations that disrupt protein homeostasis, translation, or stress-response pathways can lead to neuronal dysfunction and degeneration, providing insight into mechanisms of neurodegenerative and repeat-expansion disorders [76]. These systems allow for the direct investigation of how genetic insults interact with cellular stress pathways to influence neural viability. Recent advances have further expanded the use of organoids in neurotoxicity screening and personalized assessment. High-throughput platforms and multi-donor organoid models reveal interindividual variability in toxic responses, while co-culture systems incorporating vascular and immune components improve the physiological relevance of toxicity assays [77,78]. These studies establish brain organoids as a platform for linking diverse neurotoxic insults to cellular dysfunction and impaired neural development.

### 3.5. Neuroinflammation

Neuroinflammation plays a pivotal role in both neurodevelopmental and neurodegenerative disorders, and brain organoids enable detailed investigation of its cellular and molecular features. For cases of Leigh syndrome, there is evidence that organoids formed using Ndufs4 mutant iPSCs show an upregulated expression of inflammation mediators like NLRP3 and IL-6 coupled with microglial activation, pointing to an involvement of inflammasome activity leading to neurotoxicity [79]. For cases of metabolic diseases involving the brain, Crigler–Najjar syndromes have been shown to reproduce neuronal toxicity mediated by bilirubin through cytokine upregulation and the activation of MAPK/NF-κB signaling pathways [80]. Additionally, apart from genetic mutations and metabolic syndromes, toxic environmental factors can activate inflammation mechanisms within brain organoids. For instance, methamphetamine exposure triggers inflammatory gene up-regulation in astrocytes and neural progenitor cells, including cytokine genes, inflammasomes, and apoptotic factors [81]. Neuroinflammation in organoids is hence defined by the convergence of microglia activation, cytokines, and stress response pathways that ultimately result in neuronal dysfunction in various disease models.

### 3.6. Neuronal Deficits

The application of CO technology enables the study of the cellular mechanisms responsible for neurodevelopmental deficits caused by genetic alterations. Genetic alteration usually results in neuronal developmental defects, synaptic impairment, and excitability changes. For instance, DM1 patient-derived COs showed that the hyperphosphorylation of CELF2 caused by CUG repeat foci interferes with MECP2 regulation, disrupts excitatory synaptic signaling, causes enhanced N-methyl-D-aspartate (NMDA) receptor-mediated excitotoxicity, and leads to neuronal cell death [82]. Furthermore, the same neuronal dysfunction was found in organoids developed using 22q11.2 deletion syndrome-iPSCs, including decreased neuronal excitability and calcium signaling. The above-mentioned problems can be ameliorated by treatment with exogenous DGCR8 overexpression or pharmacological approaches, indicating the involvement of microRNA expression imbalance in developing neuropsychiatric disease [83]. Moreover, CO technology can demonstrate abnormalities on a circuit level. For instance, forebrain assembloids prepared from Timothy syndrome iPSCs showed interneuron migration defects due to altered Cav1.2 channels [84]. Neuronal dysfunction in organoids is caused by genetic manipulation affecting neuronal development, synaptic activity, and circuit integration.

### 3.7. Neuronal Activity

Neurophysiological recording and computational techniques have enhanced the possibility to study neuronal activity in brain organoids. Advanced electrophysiologic devices that support 3D recording technologies such as MEA-based platforms and penetrating electrodes allow neuronal spiking and local field potential recordings to be performed in brain organoids [85,86]. Optical modalities including light sheet imaging for calcium signal 4D acquisition make it possible to observe volumetric neuronal activities in real time, thereby contributing to the understanding of early circuit formation [87]. Computational modeling plays an important role in modeling complex neuron dynamics as well. For instance, the BCNNM framework models brain organoid development by considering neural layer formation, axon navigation, and synapse generation [88]. Functional studies demonstrate that brain organoids develop diverse neuronal subtypes, form synaptic structures, and exhibit spontaneous and stimulus-responsive activity. Patient-derived organoids modeling gray matter heterotopia display neuronal hyperactivity, altered dendritic complexity, and impaired synaptic function, linking genetic mutations to abnormal excitability [89]. In addition, optogenetic approaches enable causal interrogation of neuronal excitability and network connectivity [90]. Neuronal activity in organoids captures the emergence and modulation of neural network dynamics and their disruption in disease.

### 3.8. Neuronal Migration

Neuronal migration is a fundamental process in cortical development, essential for establishing laminar architecture and functional connectivity. COs enable investigation of the genetic and molecular mechanisms underlying this process. For instance, KIF5C, a neuron-specific kinesin, is highly expressed in early cortical neurons in both mice and human forebrain organoids, and its deficiency disrupts radial migration, dendritic branching, and spine morphogenesis, accompanied by dysregulation of genes involved in cortical projection neuron development [91]. In the same way, SYTL3 has been found to be an important regulator of brain development, where knockout experiments on brain organoids and human embryonic stem cell neurons have shown that SYTL3 is involved in regulating neuronal migration and pre-synaptic activity [92]. Mutations that lead to neurological disorders interfere with neuronal migration. Organoids derived from Fukuyama congenital muscular dystrophy patient iPSCs exhibit defective α-dystroglycan glycosylation, leading to disrupted radial glial organization and abnormal neuronal migration. The compound Mn007 shows partial rescue of this defect and emphasizes the significance of O-mannosyl glycosylation in corticogenesis [93]. In addition, organoid-based imaging systems enable dynamic analysis of migration behavior. Live imaging platforms allow real-time visualization of leading process extension, somal translocation, and migration trajectories, providing a non-invasive approach to study human neuronal migration [94]. Neuronal migration in organoids reflects the coordinated regulation of genetic programs and cellular dynamics that shape cortical organization during development.

### 3.9. Neuronal Excitotoxicity

Excitatory neurogenesis and synaptic plasticity are essential for brain development and function, with excessive glutamatergic activity leading to the excitotoxicity that contributes to neurodevelopmental and neurodegenerative disorders. In brain organoids, androgens promote excitatory neurogenesis by enhancing cortical progenitor proliferation and expanding the excitatory neuronal pool, partly through modulation of histone deacetylase activity and mTOR signaling [95]. The dual role of NMDA receptor signaling in neurotoxicity and neuroprotection is also recapitulated in human iPSC-derived organoids. Low-dose NMDA preconditioning enhances CREB phosphorylation, improves synaptic responsiveness, and activates pro-survival pathways, thereby mitigating excitotoxic injury [96]. In contrast, high-dose NMDA induces calcium overload, mitochondrial dysfunction, oxidative stress, and apoptosis, which can be attenuated by neuroprotective agents such as neutral phosphorus dendrimers [97]. In addition to ligand-mediated effects, genetic perturbations that disrupt excitatory/inhibitory (E/I) balance further exacerbate network dysfunction. Organoids carrying mutations such as PRNPE200K, trisomy 21, and LRRK2G2019S exhibit neurotransmitter imbalance, impaired glutamatergic and GABAergic signaling, and network hypersynchrony. Treatment with allopregnanolone partially restores E/I balance and improves synaptic function, indicating the potential therapeutic modulation of excitability [98]. Neuronal excitotoxicity in organoids arises from the dysregulated excitatory signaling and E/I imbalance that drive calcium-dependent stress responses and neural dysfunction.

### 3.10. Neuronal Lineage

The study of these lineage dynamics is possible due to the utilization of COs in a human-relevant model system. The analysis of lineages has shown high levels of heterogeneity of clones among neural progenitors, where subclones maintain the symmetry division capacities that ensure their expansion and adaptation under perturbations [99,100]. In addition to lineages analysis, research based on organoids has provided identification of the molecular regulators that define the fate of cells within the lineage. Functional screening approaches have indicated the genes responsible for oxidative stress response and progenitor cell survival. The discussion about clonality and adaptation also concerns the ability of neuronal lineage to differentiate into excitatory neurons, which happens mainly through intermediate progenitors, that can be transcriptionally distinct [101]. Furthermore, the disruption of neuronal lineages in COs can be explained by mutations associated with some diseases (e.g., the CTNNB1 mutation). This affects the lineage composition and gene expression [102]. On the level of systems biology, COs reproduce transcriptional programs that take place in the fetal neocortex, such as progenitor proliferation, neurogenesis, and regionalization. In addition, single-cell and epigenomic studies show conservation and unique features of human lineage regulation at the transcriptional and epigenetic levels [103,104]. Integration of clonal heterogeneity, molecular regulation, and epigenomic control is key to shaping neuronal lineage in organoids, thereby determining diversity and developmental events.

## 4. Applications of Brain Organoids

**Figure 4:**
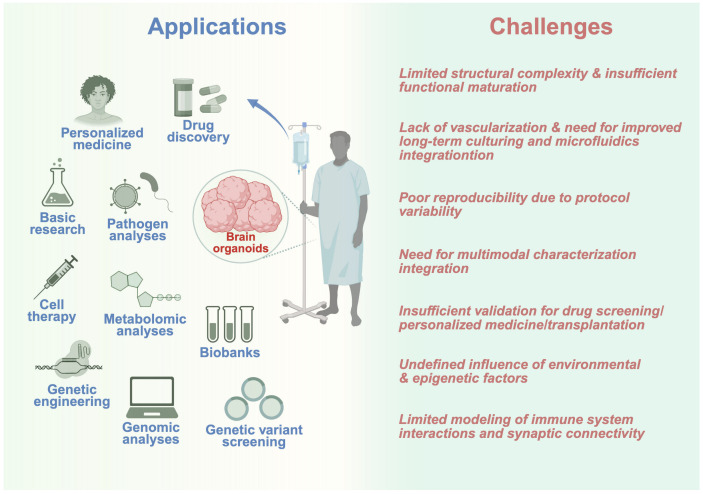
Applications and current challenges of brain organoid technology. Brain organoids represent a powerful and revolutionary tool that is applicable to various aspects of neuroscientific research from basic biology to personalized and pharmacological screenings. Among many applications, brain organoids can be used for pathogen studies, personalized medicine, drug discovery, biobanking, genetic screening and cell therapy development. However, several important challenges that should be considered while developing this technology remain unsolved. Such challenges comprise a lack of structural complexity and functional maturity, limited vascularization and long-term cultivation stability, insufficient reproducibility, and multimodal integration. Moreover, clinical translation requires thorough validation of brain organoids in addition to unknown environmental effects and incomplete knowledge of epigenomic impacts and synaptic networks.

Brain organoids represent versatile platforms that facilitate an ever-expanding spectrum of biomedical applications such as disease modeling, drug discovery, and transplantation approaches. Due to their capacity to capture both human-specific neurodevelopmental and disease phenotypes, brain organoids can undergo pharmacological analysis, multimodal studies and functional tests, including transplantation. The described applications of brain organoids are demonstrated in Figure 4.

### 4.1. Drug Screening

The use of brain organoids in drug screenings facilitates the assessment of the efficacy, toxicity, and modes of action in three-dimensional neuronal constructs. Improvements in engineering technologies like scaffold culture, microfluidic systems, and bioprinting enable better nutrient diffusion, minimize necrosis, and facilitate reproducibility, thus allowing for higher throughput and scalability of drug screenings [105,106,107]. Another advantage is provided by patient-derived organoids, such as glioblastoma organoids (GBOs). These can be utilized in the development of personalized treatment plans. Several drug screens conducted in GBO models have indicated potential targets based on specific vulnerabilities, such as TERT inhibition and proteasome dependence [108,109]. The possibility of rapid functional profiling in patient-derived brain organoids allows one to determine a personalized strategy within the clinically relevant timeframe [110]. Moreover, drug screening in brain organoids provides biomarkers that predict drug effectiveness. Recurrent GBOs differ in their sensitivity to specific targeted drugs compared with general chemotherapy due to altered pathways like Rho GTPase and NOTCH signaling [111]. In addition, mechanistically driven screening approaches have helped reveal several drug targets like DNA repair, ferroptosis, telomere maintenance, and noncoding RNA-mediated regulatory mechanisms, indicating genotype-specific vulnerabilities [112,113,114]. Drug testing on brain organoids aids in identifying therapeutic weaknesses, predicting treatment responses, and developing personalized strategies for neurological and oncological disorders.

### 4.2. Omics-Guided Disease Modeling

Omics technologies have facilitated the modeling of various diseases using brain organoids by allowing detailed molecular investigation. Multi-omics analyses combining transcriptomics, epigenomics, and proteomics identify signaling pathways and networks that regulate neural induction, differentiation, and development. Based on these findings, brain organoids recapitulate the developmental programs seen in human fetal brain tissues while also incorporating disease-specific mechanisms that could be used to refine these models [115,116,117]. On a disease level, omics-based organoid models reveal molecular characteristics of different neurological diseases. For example, glioblastoma organoids display distinct metabolic and transcriptional profiles based on their subtypes, while other models of neurological disorders, such as SCN1A-deficient organoids, have neurotransmitter dysregulation and growth impairments. Using organoid models alongside human genetic analysis can identify cell types involved in neurological diseases and potential treatments [118,119]. Nevertheless, the predictive validity of these organoid models depends on specific contexts. Although omics-based analyses of organoids tend to produce similar molecular characteristics compared with patient samples, their ability to predict disease phenotypes and treatment responses is still limited. Many organoid-based models can mimic cellular processes seen during the earlier stages of neural development while inadequately representing more advanced pathological states or systemic effects seen in patients. Single-cell analysis and computational benchmarking of organoids against primary human brain tissue can help enhance reproducibility and interpretation of omics results by identifying conserved signaling pathways and eliminating off-target cells [120,121]. Nonetheless, differences between these models and their real-life analogues persist. Differences in cellular composition, maturation state, and microenvironmental context can lead to incomplete or inconsistent representation of disease phenotypes. Moreover, while omics integration enhances mechanistic insight, successful translation of these findings into clinically validated targets or therapies remains relatively limited. In addition, comparative and co-culture studies extend these approaches to the investigation of human brain evolution and cell–cell interactions, revealing human-specific regulatory programs and immune–neural crosstalk [122,123]. Omics-guided organoid models connect molecular regulation with cell-type specificity and disease phenotypes, enabling high-resolution analysis of human brain development and pathology but should be interpreted within the constraints of their current translational and predictive limitations.

### 4.3. Organoid Transplantation

Brain organoid transplantation has emerged as an approach to study in vivo maturation, host–graft integration, and neural repair. Across multiple models, transplanted organoids demonstrate survival, vascularization, and functional integration within host tissue (Supplementary Table S1). Transplantation studies provide insight into host–graft interactions. Glia-enriched cortical organoids form astroglial networks that integrate structurally and functionally with host neurons, modulating synaptic activity and responding to inflammatory cues. The transcriptomic approach provides evidence that transplanted organoids undergo maturation processes, such as neuronal and glial development, emphasizing the impact of the cellular composition and microenvironment on transplant success [124,125]. Transplanted organoids possess the ability to connect to the recipient circuits and participate in the process of neural regeneration. In models of cortical damage, grafts form synapses with host neurons, react activity-dependently, and improve functionality. In different models of brain damage, involving both traumatic and ischemic lesions, grafts stimulate the process of neurogenesis and vascular remodeling, resulting in behavioral recovery [126,127,128]. There are many factors contributing to transplant efficiency. For instance, the stage of graft development influences its integration into the host tissue and the rate of growth. Earlier developmental stages of organoids are characterized by the capacity to grow, yet at the same time they show susceptibility to overproliferation. Molecular interventions, including modulation of survival and growth pathways, further enhance engraftment and neuronal differentiation [129,130]. Emerging strategies extend these approaches, including in vivo organoid generation and integration with neurostimulation systems to improve graft survival and functional coupling. Patient-derived organoids also enable modeling of individual-specific phenotypes and therapeutic responses [131,132]. Organoid transplantation integrates structural maturation with circuit reconstruction and functional outcomes in vivo, with graft properties and host environment jointly shaping these processes. We summarize ongoing and recently registered clinical trials incorporating brain organoids as experimental or predictive platforms (Supplementary Table S2).

Brain organoids integrate experimental modeling with translational applications across drug screening, omics-guided disease modeling, and transplantation. Drug screening approaches enable evaluation of therapeutic efficacy and identification of genotype-specific vulnerabilities. Omics technology allows for the study of the regulation of gene expression and links disease pathomechanism to cellular type, while the transplantation technique provides information about the development process, integration of circuits, and functional performance. This approach couples insights into mechanisms to drug discovery and broadens the scope of organoids in studies of neuroscience. Advances in the development of more accurate models, and incorporation into clinical data, can improve the importance of organoids in precision medicine and neuroscience.

## 5. Ethical Considerations

The development of more complex capabilities in brain organoids has created several ethical questions concerning the possibility that these organoids might possess some kind of sentience and moral standing. Research has already found that COs demonstrate spontaneous oscillations, reactivity, and, in engineered conditions, even produce muscle contractions, which are the properties of developing neural networks [133]. Several approaches, like the perturbational complexity index, have been offered to assess the degree of information integration in organoids, expanding methods for studying consciousness developed for medical purposes [134]. In addition, several theoretical approaches to COs, like the “islands of awareness” hypothesis, indicate the possible presence of some degree of fragmentation in the neural activity of organoid systems, implying that ethical implications of the complexity in biology might require revision [135]. However, ethical opinions diverge in the sense that, while some scientists state that organoids do not deserve any new kind of moral status compared with the previous models, others stress the necessity for increased supervision due to the development of new functions [136]. In general, public opinion favors brain organoid research on the condition of proper governance, accountability, and the fair distribution of benefits from organoid technology [137,138]. Specific challenges include obtaining informed consent from donors, handling genomic data ethically, and ensuring neuroprivacy [139]. These problems arise especially when applying organoid research to transplantation and personalized medicine, thus raising questions about the issues of organoids’ ownership and identity. In order to cope with these problems, precautionary approaches to ethics have been developed, including project-specific consent, ethical evaluation at each step, and the monitoring of threshold points of functional complexity [140,141]. It is essential to develop frameworks for the regulation of the commercialization and creation of chimeras, along with equitable access to novel technologies.

## 6. Limitations and Future Perspectives

The rapid advancement in brain organoid technologies has expanded opportunities in the development of in vitro models of human brain development, disease pathogenesis, and therapeutic approaches. A few key issues need to be addressed for more widespread use of organoid technology in research. Improvement of structural complexity and functional maturity through vascularization, culturing and interfacing with engineering systems would increase their ability to mimic physiological environments. Despite the significant progress achieved in the field, the successful establishment of the functional and perfused vasculature system remains an open challenge. Different approaches, like vascularization via co-culturing endothelial cells, induction of vascular structures, and in vivo transplantation of organoids, demonstrate some level of vascularization, but fail to recreate many of the physiological properties of the brain vasculature, like the hierarchical organization of vessels, perfusion, and neurovascular coupling. The failure to establish the vascular system is driven by the absence of the systemic circulation, the lack of adequate biomechanical stimulation (shear stress), and the insufficient presence of the pericytes and astrocytes required for further vascularization. Diffusion limits thus remain an issue for oxygen and nutrient supply in vitro, restricting further growth of tissues and the ability to achieve complete maturation of organoids. Establishment of standards of cell sources, differentiation protocols, and culturing conditions would help in making results more comparable across laboratories. The utilization of multimodal approaches such as single-cell omics analysis, electrophysiology, and live imaging would further improve the resolution of obtained results. At the same time, creation of patient-specific brain organoids combined with genome editing tools would create unique opportunities for precision medicine for neurological and neuropsychiatric disorders. The key issues to be solved include, among others, incomplete modeling of immune responses, limited neuronal connectivity, and variability across different systems.

From a more fundamental perspective, several important challenges still prevent brain organoids from achieving their full potential as accurate and reliable models of human brain physiology. The first involves the inability of current brain organoids to reproduce circuit-level organization due to the lack of proper afferent and efferent connectivity and interactions between distant brain regions. This is especially relevant when talking about the modeling of subcortical systems and the broader brain-wide integration necessary to study more complex neurological and neuropsychiatric disorders. Additionally, most organoid cultures resemble early developmental stages of brain physiology, which leads to difficulties with modeling adult-onset diseases characterized by different sets of phenotypes. Due to the incomplete maturation of cells and neural circuits, brain organoids cannot fully model the complex pathology associated with later stages of life. Inconsistency in results and reproducibility due to variability in cell sources, differentiation protocols, and lab conditions represents another major issue for the field. Finally, interpreting organoid electrophysiology may represent additional challenges as electrical activity measured in brain organoids does not necessarily correspond to physiological behavior and circuitry. These limitations suggest that, though brain organoids present promising model systems, they are as yet unable to accurately replicate circuit-level and clinically relevant physiological behavior. In addition, the focus on ethical issues, especially those linked with greater functional complexity, will continue alongside the move toward translational applications. Further advancements will depend on a combination of developments in bioengineering, data integration, and standardization of experiments, making brain organoid models more biologically relevant and applicable to translation.

## 7. Conclusions

Advances in brain organoids as models of human brain development, disease mechanisms, and therapies have been well documented. By harnessing advancements in areas of stem cell biology, bioengineering, and multi-omics approaches, these systems allow studies in the field ranging from the level of organization to function. Improved control of patterning and growth through engineered culturing systems allows functional studies that shed light on the phenomena of neurogenesis, circuit function, and disease-related changes that can inform studies in drug discovery, omics-based disease modeling, and transplantation therapy. Challenges that still exist in this field include limited maturity, lack of connectivity, variation between different techniques used, and modeling of system-level interactions. Ethical issues pertaining to the increased functionality of organoids need to be addressed as well. Future advancements in the fields of standardization, multimodality, and alignment with human datasets are expected to augment the biological significance and translational capabilities of organoid platforms. Collectively, brain organoids can be considered an umbrella approach that connects developmental biology, disease modeling, and therapeutic research in human neuroscience.

## Data Availability

No new data were created or analyzed in this study.

## Supplementary Materials

The following supporting information can be downloaded at https://www.mdpi.com/article/10.3390/brainsci16040427/s1, Table S1: Summary of brain organoid transplantation; Table S2: Summary of brain organoid-based clinical trails.
