# Supplementary material for: Brain Organoids: Emerging Platforms for Modern Neuroscience
Source: Brain Sci. 2026 Apr 19;16(4):427. doi: 10.3390/brainsci16040427 (PMC13114865; doi:10.3390/brainsci16040427)
Supplement: Supplementary file 1 [file brainsci-16-00427-s001.zip › brainsci-4238251-supplementary.pdf]

Supplementary Table S1. Summary of brain organoid transplantation

Organoid Type	Transplantation Site	Host Species	Host Model	Findings & Mechanism	Refer.
hPSC-derived glia-enriched cortical organoids	dorsolateral hippocampus	NOD-SCID mouse	Healthy	Transplanted organoids exhibited regional astrocyte diversification, mature astrocyte morphology, and immune reactivity via TNFα-induced CD38 signaling	124
hESC/hiPSC derived brain organoids	Prefrontal cortex & hippocampus	SCID mouse	Healthy	Transplanted cells exhibited sequential neural and glial differentiation—neurogenesis at 2 months followed by glial maturation at 4 months, with more mature astrocytes in the PFC than in the hippocampus, modulated by region-specific dopamine- and acetylcholine-dependent calcium signaling.	125
hiPSC-derived cortical organoids	Somatosensory cortex	Athymic rat	Healthy	Transplanted human cortical organoids matured and integrated into thalamocortical and reward-related circuits, received sensory input, extended long-range axons, and modulated reward-seeking behavior via optogenetic stimulation, supported by activity-dependent transcription and synaptic/membrane maturation.	126
hESC-derived cerebral organoids	Damaged motor cortex	Sprague-Dawley Rat	TBI	55-day organoids exhibited superior survival, neurogenesis, and reduced apoptosis/inflammation compared to 85-day organoids, enabling multilineage differentiation, motor cortex reconstruction, neurotrophic factor upregulation, and migration along the corpus callosum to promote motor recovery.	127
hESC/hiPSC derived brain organoids	Junction of infarct core and peri-infarct zone	NOD-SCID Mouse	Photothrombotic stroke model	Transplanted organoids survived, differentiated into neurons, and reconstructed infarcted cortex via region-specific repair, axonal projection, and integration into host circuits, thereby improving sensorimotor function—unlike dissociated cells, which failed to repair.	128

hiPSC-derived cerebral organoids	Frontal motor cortex	SCID Mouse	TBI	Progranulin pretreatment enhanced survival, engraftment, and corticospinal axon extension of hiPSC-COs via Akt phosphorylation, increasing subcerebral projection neurons for reconstructing injured circuits.	129
hESC-derived cerebral organoids (6w & 10w)	Frontal/parietal cortex (mouse), precentral gyrus (monkey)	SCID Mouse; cynomolgus Monkey	Cerebral cortex injury model	Transplanted cerebral organoids showed developmental stage-dependent axonal growth, with mature 10w-organoids exhibiting enhanced corticospinal projections and reduced overgrowth, enabling successful engraftment and connectivity in both mouse and primate brains post-injury.	130
hPSC-derived premature cerebral organoids	Striatum	SCID Mouse	Healthy	In vivo–developed organoids generated diverse neural subtypes, including striatal neurons, and exhibited enhanced supporting cell development with reduced cellular stress and apoptosis compared to in vitro counterparts.	131

Akt, protein kinase B; CD38, cluster of differentiation 38; COs, cortical organoids; hESC, human embryonic stem cell; hiPSC, human induced pluripotent stem cell; hPSC, human pluripotent stem cell; NOD-SCID, non-obese diabetic severe combined immunodeficiency; PFC, prefrontal cortex; SCID, severe combined immunodeficiency; TBI, traumatic brain injury; TNFα, tumor necrosis factor-alpha.

Supplementary Table 2. Summary of brain organoid-based clinical trails

Disease	Model System	Methodology	Main Goal	Relevance to Organoids	ClinicalTrials.gov ID	Study Start	Status
Glioblastoma	GBOs + BVO from perioperative samples	Ex-vivo organoid culture and phenotypic/molecular analysis	Feasibility of GBO/BVO generation in routine clinical practice and molecular profiling of the glio-vascular niche	Brain organoids applied to characterize tumor–vascular interactions and link organoid phenotypes to clinical outcomes	NCT07029100	2025-07-01	Not yet recruiting
BD	Cortical organoids derived from iPSC (from patient PBMCs)	Blood sampling, iPSC generation and cortical organoid differentiation	Identify early neurodevelopmental alterations in BD patients with vs without neurodevelopmental burden	Brain organoids used to dissect disease-specific neurodevelopmental signatures underlying psychiatric disorders	NCT06968598	2025-06-01	Not yet recruiting
GBM	PDOs and GSCs	Organoid culture, drug screening, epigenetic & splicing modulation	Discover immunogenic neoepitopes and therapeutic vulnerabilities in GBM	PDOs used to model tumor heterogeneity and predict treatment response	NCT06781372	2025-04-01	Not yet recruiting
Breast cancer brain metastases	PDO from brain or extra-cranial metastases	PDO drug/radiotherapy sensitivity, IC50, immune co-culture	Predict patient outcomes based on PDO response to treatment	Use of PDOs to assess therapy response and correlate with clinical prognosis	NCT06468124	2024-07-01	Not yet recruiting
Pediatric brain tumors (and other pediatric solid tumors)	iPSC-derived immune organoids with	iPSC reprogramming, immune-organoid engineering, tumor engraftment	Study tumor-immune interactions in pediatric cancers	Develop immune-integrated organoid models for therapeutic testing	NCT05890781	2023-05-12	Recruiting

Brain tumors and neurological diseases	patient tumor tissue hiPSC-derived cerebral organoids & human surgical brain tissue	Optical microscopy + electrophysiological readouts	Build image/signal database; compare organoid vs human brain	Validate fidelity of organoids in clinical modeling	NCT05921786	2023-05-01	Recruiting
Recurrent high-grade astrocytic glioma	PDO + QPOP-guided therapy	PDO generation, QPOP drug screening, MRI biomarker analysis	Optimize individualized drug combinations and assess therapy response	PDOs used to match ex vivo drug sensitivity with patient outcomes	NCT05532397	2023-02-17	Recruiting
Low-grade and high-grade glioma	Patient-derived glioma organoids (LGG/HGG PDOs) from surgical tissue	Phenotypic, genetic, epigenetic, proteomic, transcriptomic profiling; co-culture with immune cells; treatment testing (chemo/IO ± radiation)	Establish a living organoid biobank and dissect mechanisms of resistance, dedifferentiation and invasion	Brain organoids serve as patient avatars to model malignant behavior and evaluate combinational treatment strategies	NCT04865315	2022-05-01	Recruiting
Glioblastoma (primary/recurrent)	GBOs + Drug Sensitivity Test	GBO establishment, drug screening (TMZ + repurposed drugs), IC50 vs. PFS/OS correlation	Validate organoid-based prediction of drug response	GBOs used to test drug response & correlate with clinical outcomes	NCT06782984	2021-08-18	Recruiting
Glioblastoma	GSC-derived organoids from GBM surgical tissue	Organoid modeling, MGMT methylation profiling, drug resistance testing, ctDNA analysis	Study TMZ resistance and identify treatment-persistent clones	GSC organoids model intratumoral heterogeneity and therapy-induced evolution	NCT04868396	2021-04-10	Active, not recruiting

Vestibular schwannoma	Schwannoma stem cell organoids from surgical tumour tissue	Feasibility of organoid generation and profiling (phenotype/genetic/epigenetic/proteomic/transcriptomic)	Establish a living biobank of vestibular schwannoma organoids to study resistance mechanisms	Organoids enable mechanistic characterization and potential treatment stratification of cranial nerve sheath tumors	NCT05786144	2020-06-01	Recruiting
High-grade astrocytoma	hiPSC-derived brain organoids with glioma-related mutations	iPSC reprogramming, gene editing, tumor modeling, IHC	Model gliomagenesis using patient-specific genetic mutations	Establish brain organoids as a platform to study early tumor initiation	NCT03971812	2019-06-07	Unknown status
Glioblastoma, Malignant Glioma	Autologous iPSC-derived brain organoids + patient-derived GSCs	Organoid-GSC co-culture, gene modulation, 3D genome profiling	Analyze GSC invasion & identify anti-invasion/ciliogenesis strategies	Personalized organoid platform to test tumor invasion and gene function	NCT05772741	2018-12-03	Unknown status

BD, Bipolar disorder; BVO, Blood vessel organoid; ctDNA, Circulating tumor DNA; GBM, Glioblastoma multiforme; GBO(s), Glioblastoma organoid(s); GSCs, Glioblastoma stem-like cells; HGG, High-grade glioma; hiPSC, Human induced pluripotent stem cell; IHC, Immunohistochemistry; IO, Immuno-oncology; iPSC, Induced pluripotent stem cell; LGG, Low-grade glioma; MGMT, O-6-methylguanine-DNA methyltransferase; MRI, Magnetic resonance imaging; OS, Overall survival; PBMCs, Peripheral blood mononuclear cells; PDO, Patient-derived organoid; PFS, Progression-free survival; QPOP, Quadratic phenotypic optimization platform; TMZ, Temozolomide.
